# Lynch syndrome with *MLH1* germline variant in an extended family: a case report

**DOI:** 10.1515/biol-2025-1275

**Published:** 2026-04-20

**Authors:** Zhipei Duan, Chengru Hu, Tinghua Cao, Chao Chen, Yan Li

**Affiliations:** Department of Oncology, Suzhou Ninth People’s Hospital, Suzhou Ninth Hospital Affiliated to Soochow University, Suzhou 215200, China

**Keywords:** Lynch syndrome, colon cancer, *MLH1*, germline variant, case report

## Abstract

Lynch syndrome (LS) is an inherited cancer predisposition syndrome associated with an increased risk of several malignancies, particularly colorectal cancer (CRC). The diagnosis of LS is typically based on family history and confirmed by genetic testing, most commonly involving pathogenic variants in mismatch repair genes, including *MLH1*, *MSH2*, *MSH6*, and *PMS2*. We report a proband who developed CRC at the age of 52 years and had a family history suggestive of LS. Immunohistochemical analysis revealed loss of MLH1 and PMS2 expression in the proband, and isolated loss of PMS2 expression in his maternal cousin. Whole-exome sequencing followed by Sanger sequencing identified a heterozygous *MLH1* c.1652A>C (p.Asn551Thr) variant in the proband, his maternal cousin, and four additional affected family members. Based on the clinical, pathological, and genetic findings, the extended family was diagnosed with LS. Taken together, *MLH1* c.1652A>C (p.N551T) variant may contribute to carcinogenesis, and its co-segregation with LS in this family provides supportive evidence for its potential pathogenicity.

## 1 Introduction

The majority of colon cancer (CC) cases are sporadic, arising from complex interactions among environmental exposures, lifestyle factors, and somatic mutations accumulated over an individual’s lifetime [1]. The remaining cases have an inherited component and are typically identified through familial clustering of specific cancer types or characteristic cancer spectra [2]. One such inherited cancer syndrome is exemplified by the observations of Lynch and colleagues, who in 1966 described extended pedigrees with a high incidence of CC in conjunction with gastric and endometrial cancers [3], 4]. This syndrome, later termed Lynch syndrome (LS) or hereditary nonpolyposis colorectal cancer (HNPCC), is a genetic condition associated with a markedly increased risk of multiple malignancies [5], [6], [7]. It is estimated to affect approximately 1 in 300–400 individuals in the general population, making it one of the most common hereditary cancer syndromes [8].

In LS, autosomal dominant heterozygous germline variants in mismatch repair (MMR) genes lead to loss of protein function and confer a markedly increased predisposition to malignancy [9]. Compared with sporadic CC, LS typically occurs at a younger age and shows strong familial clustering [10]. Accordingly, regular surveillance, including colonoscopic screening and other preventive strategies, is recommended to enable early detection and timely management [11]. Genetic testing and counseling are therefore essential for individuals with a family history suggestive of LS, both for risk stratification and for guiding preventive interventions.

Although clinical criteria and tumour-based screening are widely used in clinical practice to screen for LS, a definitive diagnosis is established through germline genetic testing identifying pathogenic or likely pathogenic variants in MMR genes [12]. Genetic testing typically involves analysis of mutL homologue 1 (*MLH1*), mutS homologue 2 (*MSH2*), *MSH6*, or postmeiotic segregation increased 2 (*PMS2*) [13]. In addition, deletions involving the epithelial cell adhesion molecule (EPCAM) can result in epigenetic silencing of *MSH2* and are therefore included in many diagnostic testing panels [11]. Copy number variations (CNVs), including large deletions or duplications, also contribute to LS and are assessed according to the NCCN guidelines [14].

Despite increasing recognition of LS, the full spectrum of pathogenic germline variants, particularly rare or previously unreported *MLH1* variants, remains incompletely defined. This gap limits our ability to refine genotype–phenotype correlations in affected families. Herein, we report a family with LS in whom we identified a germline *MLH1* variant, c.1652A>C (p.N551T), thereby contributing additional evidence to the expanding the pathogenesis of LS.

## 2 Case presentation

**Figure 1:**
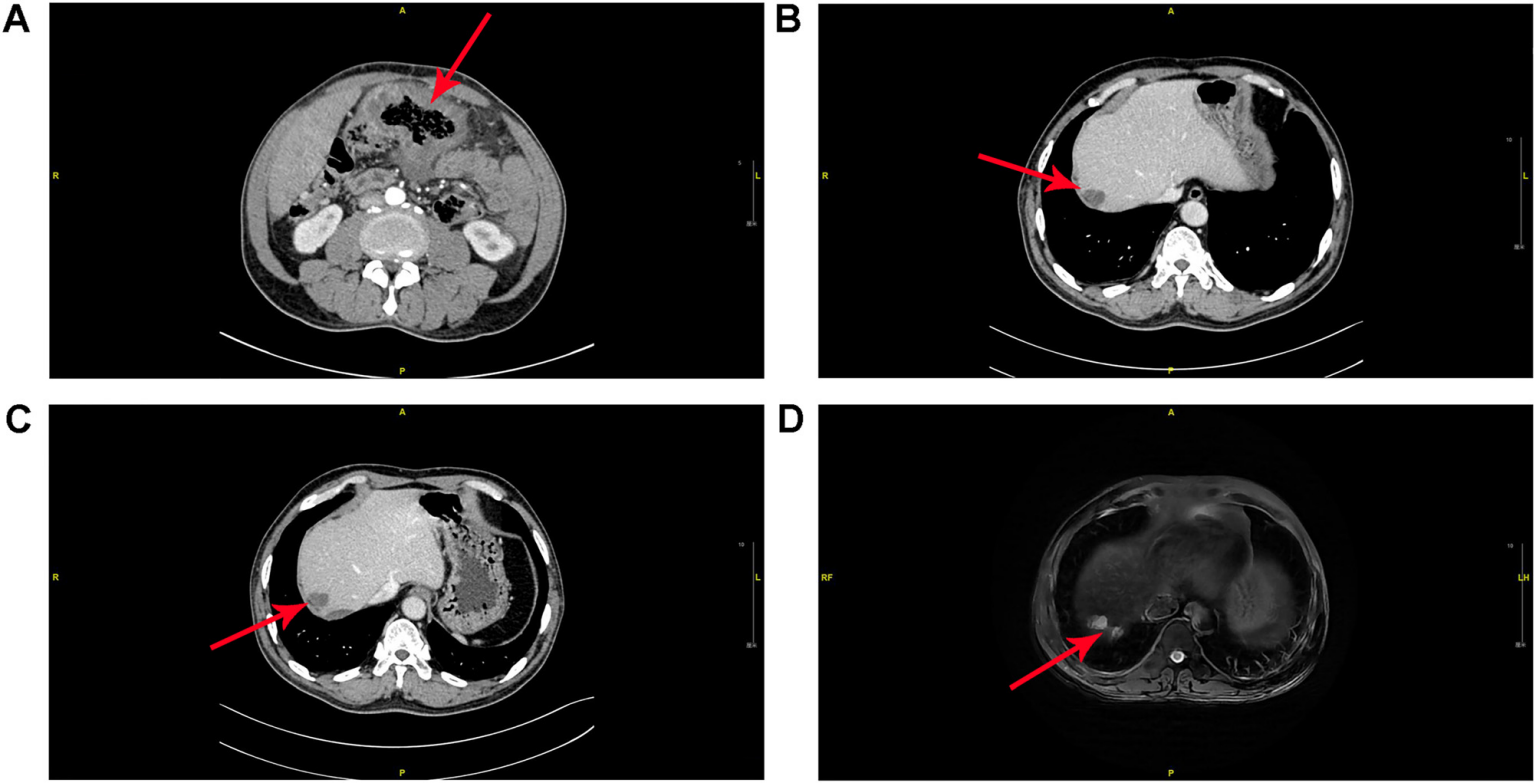
Computed tomography (CT) scan of the abdomen performed on admission and MR images of the upper abdomen during the postoperative follow-up. (A) Preoperative CT indicated marked dilatation from the hepatic flexure to the transverse colon with associated bowel wall thickening (red arrow), raising suspicion for colon cancer. (B) In the 10 months follow-up, CT scan indicated a subcapsular low-density lesion (red arrow) in the right hepatic lobe, consistent with suspected hepatic metastasis. (C) After sintilimab-based immunotherapy, the patient showed no significant progression (red arrow) at 22 months compared with the 10-month follow-up. (D) On T2-weighted imaging, the metastatic lesion in the right hepatic lobe demonstrates mildly increased signal intensity with a slight interval increase in size (red arrow), as revealed on upper abdominal MR.

A 52-year-old gentleman presented to our hospital in 2021 with frequent abdominal pain. Routine blood tests revealed elevated carcinoembryonic antigen (CEA, 20.35 ng/mL; normal 0.1–5.0 ng/mL) and carbohydrate antigen 19–9 (CA199, 124.0 U/mL; normal 0.2–37.0 U/mL). Contrast-enhanced computed tomography (CT) of the chest and abdomen demonstrated a mass involving the hepatic flexure and transverse colon (Figure 1A). The patient subsequently underwent extended right hemicolectomy (ERH). Pathological examination confirmed ulcerative mucinous adenocarcinoma of the right hemi-colon with full-thickness invasion.

**Figure 2:**
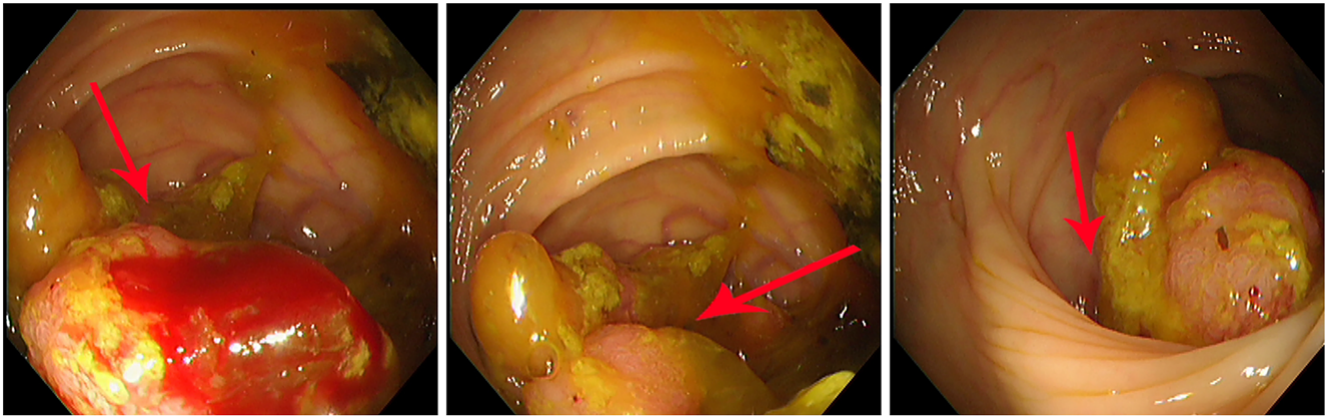
Enteroscopy showed a large neoplasm in the transverse colon close to the hepatic flexure. (A) The lesion was friable with bleeding (red arrow) after biopsy. (B) A large neoplastic lesion (red arrow) was identified at the hepatic flexure of the transverse colon. (C) The lesion showed luminal narrowing (red arrow).

Adjuvant chemotherapy was initiated approximately one month after surgery and completed over a five-month period. At approximately 10 months of follow-up, abdominal CT revealed suspected metastatic lesions (Figure 1B). First-line therapy with bevacizumab, oxaliplatin, and capecitabine was initiated, but due to poor tolerance, the regimen was switched to sintilimab-based immunotherapy. Follow-up CT at approximately 22 months revealed no significant progression of liver lesions (Figure 1C). However, magnetic resonance imaging (MRI) at 31 months showed multiple abnormal liver signals suspicious for metastasis (Figure 1D). Enteroscopy revealed a friable, bleeding mass in the transverse colon with associated stenosis (Figure 2).

**Figure 3:**
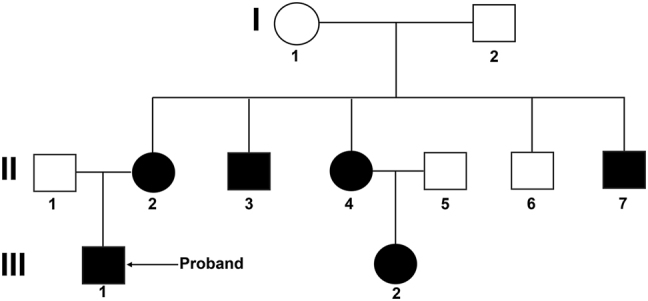
Pedigree of a family with LS. The diagnosis of LS was fully consistent with the Amsterdam II diagnostic criteria. The arrow indicates the proband. Circles and squares represent females and males, respectively. Black-filled symbols indicate affected individuals. All the affected relatives all carried the *MLH1* c.1652A>C (p.N551T) variant.

The patient reported a notable family history of CC spanning three generations as shown in Figure 3. His mother (II-2) had well-differentiated adenocarcinoma of the descending colon (Stage I). His maternal uncles (II-3 and II-6) and maternal aunt (II-4) were diagnosed with moderately or well-differentiated colon adenocarcinoma (Stages II–III). A maternal cousin (III-2) also had moderately differentiated colon adenocarcinoma.

**Figure 4:**
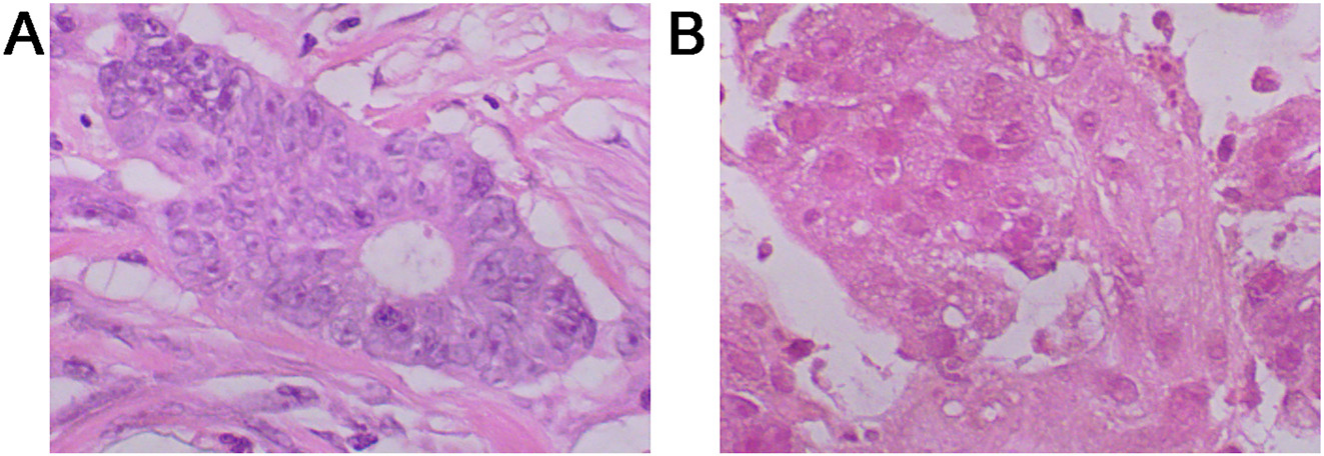
Representative images of H&E staining. (A) HE staining of malignant colon tumor cells from III-1. The malignant cells show infiltrative growth with marked cytologic atypia. (B) HE staining of malignant colon tumor cells from III-2. The malignant cells are diffusely arranged, exhibiting pleomorphism with variable cell size and shape, enlarged irregular nuclei, and hyperchromasia. The images were observed under a magnification of 400×.

**Figure 5:**
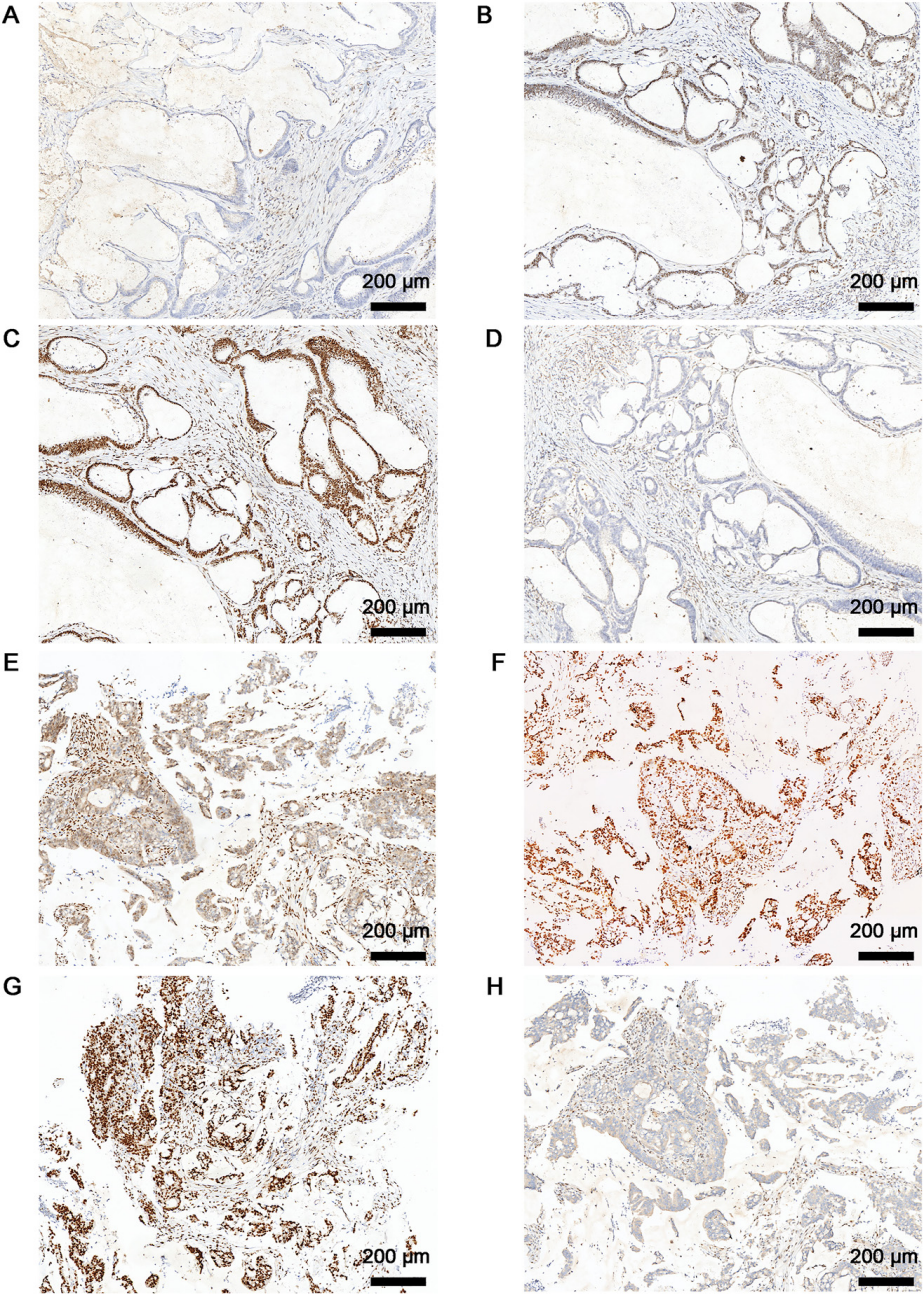
IHC staining of MMR proteins in CC tissues. Representative images of MLH1 (A, E), MSH2 (B, F), MSH6 (C, G) and PMS2 (D, H) from III-1 and III-2. The images were obtained under a magnification of 10×.

For the histopathological evaluation, hematoxylin and eosin (H&E) staining was performed for the proband and his maternal cousin (Figure 4A and B). Immunohistochemistry (IHC) staining results of the proband (III-1) were as follows: MLH1 (−) (Figure 5A), MSH2 (+) (Figure 5B), MSH6 (+) (Figure 5C), and PMS2 (−) (Figure 5D). IHC analysis for III-2 showed positive staining of MLH1 (+) (Figure 5E), MSH2 (+) (Figure 5F), MSH6 (+) (Figure 5G), and negative staining of PMS2 (−) (Figure 5H).

**Figure 6:**
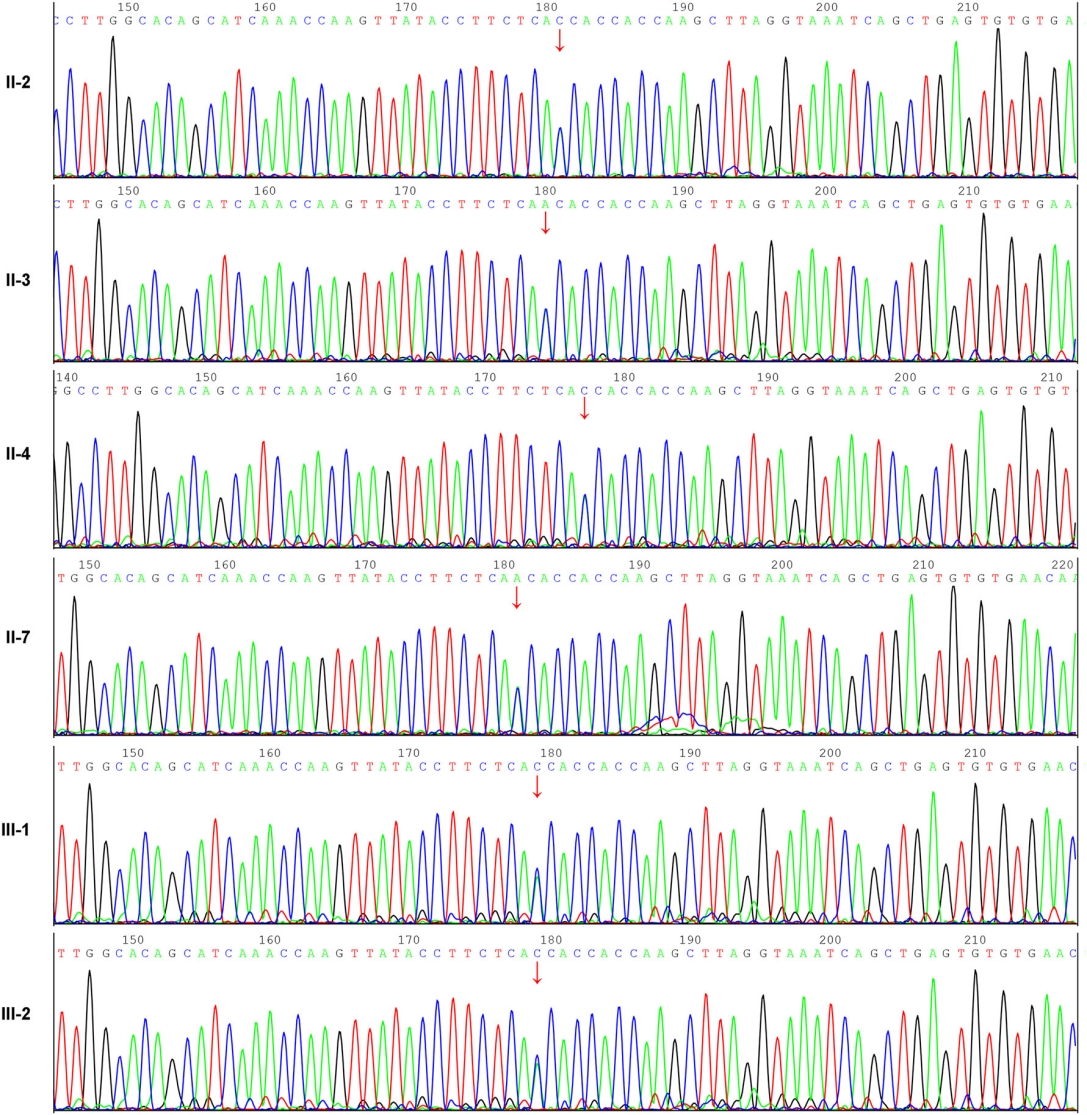
Sanger sequencing results for the six patients. Red arrows indicate the mutation sites.

Whole-exome sequencing (WES) followed by Sanger validation was performed on peripheral blood samples after informed consent. The proband, his maternal cousin, and four other affected relatives all carried the *MLH1* c.1652A>C (p.N551T) variant, thus this variant demonstrated co-segregation with CC within the family (Figure 6). These findings are consistent with the *MLH1* c.1652A>C (p.N551T) variant being causal of the LS phenotype in this family.

For the treatment, all affected members underwent surgical resection, and some received chemotherapy (II-4, II-6) or immunotherapy (II-3, III-1). All the patients were still alive at last follow-up in January 2024.

**Informed consent:** Informed consent has been obtained from all individuals included in this study.

**Ethical approval:** The research related to human use has been complied with all the relevant national regulations, institutional policies and in accordance with the tenets of the Helsinki Declaration, and has been approved by the Ethical Committee of Suzhou Ninth People’s Hospital (Approval No.: KYLW2024-005-01).

## 3 Discussion

LS is an autosomal dominant hereditary cancer predisposition syndrome characterized by an increased risk of colorectal and endometrial cancers, among others, resulting from inherited pathogenic variants in MMR genes [15], 16]. The *MLH1* c.1652A>C (p.N551T) variant has been previously reported in LS–affected families and is recorded in publicly accessible databases such as the InSiGHT-curated LOVD [17], 18]. For instance, Hutter et al. published one case in a Swiss family meeting Amsterdam I criteria, with 4 family members affected and carrying this variant [19]. Our report adds further evidence by describing an additional family of distinct ethnic background showing co-segregation of this variant with disease.

LS typically develops at a younger age than sporadic CC, with a reported mean age of onset ranging from 45 to 60 years compared with approximately 69 years in sporadic cases, although variation exists depending on the specific genotype [9]. In the present study, cancer onset occurred before the age of 60 years in all affected individuals except the proband’s mother, who was diagnosed at 67 years of age. Consistent with previous reports, tumors predominantly arose in the proximal colon, including the ascending and transverse colon, a distribution distinct from sporadic CC, which more commonly involves the distal colon [20]. In addition, individuals with LS are at increased risk of developing synchronous or metachronous colorectal tumors [21]. Histopathologically, LS-associated tumors frequently exhibit mucinous differentiation, poor differentiation, or medullary growth patterns, along with prominent tumor-infiltrating lymphocytes reflecting a high neoantigen load secondary to microsatellite instability [22], 23]. In the present family, three individuals (II-2, II-4, and II-7) were diagnosed with moderately to well-differentiated adenocarcinomas with T2–T4 invasion and no lymph node metastasis. Two patients (II-3 and III-2) presented with moderately differentiated mucinous adenocarcinomas involving multiple colonic segments. At the most recent follow-up, none of these cases showed evidence of distant metastasis. In contrast, the proband was diagnosed with ulcerative mucinous adenocarcinoma of the right hemi-colon with full-thickness invasion and developed liver metastasis within 8 months after surgery, suggesting inter-individual heterogeneity in disease aggressiveness despite shared genetic background.

For the diagnosis of LS, traditional clinical screening tools, such as the Amsterdam II and revised Bethesda criteria, are limited by relatively low sensitivity when used alone [24]. Consequently, universal tumor screening using microsatellite instability (MSI) testing and IHC for MMR proteins has been widely adopted. When concurrent loss of MLH1 and PMS2 is detected by IHC, further evaluation using *BRAF* testing and *MLH1* promoter methylation analysis is recommended to distinguish sporadic cases from hereditary LS. Loss of MLH1 function often leads to secondary degradation of PMS2, explaining the frequent combined loss of these proteins [25]. In our study, the observed IHC patterns were consistent with underlying MLH1 dysfunction. Notably, retained MLH1 expression in one heterozygous carrier (III-2) may reflect expression from the wild-type allele, highlighting the complexity of IHC interpretation in LS diagnostics.

Limited data are available regarding the *MLH1* c.1652A>C (p.N551T) variant. This residue lies within a region implicated in interaction with PMS2, suggesting potential functional relevance [26]. *MLH1* c.1652A>C substitution may impair MLH1 expression and MMR activity, supporting its pathogenic role in LS [27]. LS-associated *MLH1* variants have been shown to result in marked inter-tumoral variability in MSI targets, including transforming factor *β* receptor II (TGFβRII), BAX, CASPASE-1 (CASP1), and amyloid precursor protein (APP) [28]. Although several reports support the pathogenicity of the *MLH1* c.1652A>C variant [26], 29], 30], other studies have yielded inconclusive results, noting a lack of statistically significant structural impact on protein function and its occasional presence in individuals without LS-associated malignancies [31]. These discrepancies underscore the importance of integrating functional data, co-segregation analysis, and detailed phenotypic characterization when interpreting rare *MLH1* variants.

LS typically manifests in families with multiple affected members across generations, enabling identification of individuals at high lifetime cancer risk and facilitating targeted surveillance and preventive strategies. In this family, the *MLH1* c.1652A>C (p.N551T) variant co-segregated with disease across several affected relatives, supporting its clinical relevance. Although universal genetic testing is not cost-effective for all CC patients, germline testing should be strongly considered in individuals with early-onset disease, proximal tumor location, MMR deficiency, or a suggestive family history. Recently, immunotherapy, particularly PD-1 blockage, has shown promising results in treating LS [32]. Additionally, immune checkpoint inhibitors can induce durable responses in patients with the LS who have metastatic CC [33]. In this study, both the proband and one maternal uncle received immunotherapy and demonstrated favorable clinical responses, further supporting the efficacy of immune checkpoint blockade in LS.

There are some limitations in this case report. One limitation is that unaffected relatives were not evaluated clinically and therefore were not included in germline sequencing, which precludes assessment of non-carrier status and age distribution among unaffected individuals. Although *MLH1* c.1652A>C (p.N551T) has been reported in multiple families meeting Amsterdam criteria, functional evidence supporting a definitive pathogenic role remains limited, and the variant is currently classified as a variant of uncertain significance and no consensus on pathogenicity. While our report contributes additional clinical and familial data from a Chinese family, thereby expanding the ethnic spectrum associated with this variant, it does not in isolation provide sufficient evidence to support definitive reclassification.

## 4 Conclusions

We report a Chinese family with clinical features consistent with LS carrying the *MLH1* c.1652A>C (p.N551T) variant. Our findings add further clinical and familial evidence supporting a potential association between this variant and LS, expanding its reported ethnic spectrum.
